# The involvement of high succinylation modification in the development of prostate cancer

**DOI:** 10.3389/fonc.2022.1034605

**Published:** 2022-11-01

**Authors:** Zhenyang Zhang, Yanru Chen, Lingyu Fang, Jiang Zhao, Shishan Deng

**Affiliations:** ^1^ Institute of Basic Medicine and Forensic Medicine, North Sichuan Medical College, Nanchong, Sichuan, China; ^2^ Breast Cancer Biological Targeting Institute, North Sichuan Medical College, Nanchong, Sichuan, China; ^3^ Department of Urology, Second Affiliated Hospital, Army Military Medical University, Chongqing, China

**Keywords:** prostate cancer, succinylation, post-translational modification, GLYATL1, PDL1

## Abstract

**Objective:**

Succinylation modification of the lysine site plays an important role in tumorigenesis and development, but it is rarely reported in prostate cancer (PCa), so this study aims to elucidate its expression in and clinical correlation with PCa.

**Methods:**

A total of 95 tumor, 3 normal and 52 paired adjacent tissue of PCa were involved for succinylation stanning. 498 PCa samples with 20 succinylation modification-related genes from TCGA were downloaded for model construction. Statistical methods were employed to analyze the data, including Non-Negative Matrix Factorization (NMF) algorithm, t-Distributed Stochastic Neighbor Embedding (t-SNE) algorithm and Cox regression analysis.

**Results:**

The pan-succinyllysine antibody stanning indicated that tumor tissues showed higher succinyllysine level than adjacent tissues (p<0.001). Gleason grade and PDL1 expression levels were significantly different (p<0.001) among the high, medium and low succinylation staining scores. The types of PCa tissue were divided into four clusters using RNA-seq data of 20 succinylation-related genes in TCGA database. Clinical characterize of age, PSA level, and pathological stage showed differences among four clusters. The expression of succinylation-related genes (KAT5, SDHD and GLYATL1) and PCa related genes (PDL1, AR and TP53) were significantly different in 52 matched tumor and adjacent tissues (p<0.001). GLYATL1 and AR gene expression was significantly related to the pathological stage of PCa.

**Conclusion:**

Succinylation was significantly increased in PCa tissues and was closely related to Gleason grade and PD-L1 expression. Model construction of 20 genes related to succinylation modification showed that the later the pathological stage of PCa, the higher the level of succinylation modification.

## Introduction

The GLOBOCAN database of the International Center for Research on Cancer (IARC) showed that 1,414,259 new cases of prostate cancer (PCa) emerged in 2020, in which 375,304 were dead, marking the cancer as the fourth most common in the world and the second most common among men (1). Most PCa is found at an early stage, so the treatment effect is good. However, about 10% of PCa patients are diagnosed with metastasis with poor prognosis and a 5-year survival rate of about 30% (1, 2). Early PCa can be treated by active surveillance, endocrine, surgery, radiation therapy and other means (2–4). Androgen-deprivation therapy (ADT) is the main treatment and is usually used in various stages, from early local diseases to late distant metastatic diseases. It has been reported that 10%-20% of PCa patients are resistant to ADT and eventually develop into castration-resistant prostate cancer (CRPC), and the prognosis is poor (4–7). Studies have found that androgen receptor (AR) signaling is related to the underlying molecular mechanism of CRPC under castration, but the specific mechanism is still unclear, and there is currently no effective clinical treatment for CRPC (4–7). Therefore, it is of great clinical significance to explore the mechanism of the occurrence and development of prostate cancer, find new key markers, and provide new ideas for clinical diagnosis and treatment. Studies suggest that amino acids, carnitine derivatives, PSA density, and multiparameter MRI have been used for prostate cancer prediction and prognosis analysis (4–7). However, there are few reports about the role and value of succinylation modification of lysine sites in prostate cancer.

Protein post-translational modification (PTM) is ubiquitous in various prokaryotes and eukaryotes, participating in various life activities (8). Common PTMs include phosphorylation, acetylation, methylation, ubiquitination, methylation malonylation, and succinylation. Because succinylation modification is relatively conservative in biological evolution, it involves almost all biological processes of organisms (8). Therefore, compared with other PTMs, succinylation can induce more changes in protein physicochemical properties and functions (8). With the continuous development of mass spectrometry technology, studies have found that succinylation is involved in many biological processes, such as oxidation, metabolic regulation, signal transduction, etc., and is closely related to the occurrence and development of various human diseases (9). In thyroid, breast, lung and colon cancers, succinylation promotes tumor proliferation, differentiation and metastasis through histone and non-histone modification (10). However, there are few reports on the clinical correlation between succinylation level and PCa. In the current study, we detected the expression level of succinylation in PCa tissues through immunohistochemistry. The RNA sequence data published by TCGA are used to verify the key role of succinylation in the development of PCa, which may provide a new strategy for the diagnosis and treatment of PCa.

## Methods

### Clinical Samples

95 cases of PCa, 52 cases of adjacent tissues, and 3 cases of normal prostate paraffin-embedded tissues were collected from the Second Affiliated Hospital of the Army Military Medical University from January 2018 to March 2022. All patients with PCa underwent radical prostatectomy treatment in our hospital; none received radiotherapy, chemotherapy, or anti-androgen before surgery. All tissues were diagnosed by more than 2 pathologists after routine HE staining. The age of the 95 PCa patients ranged from 51 to 84 years, with a mean age of (70.7 ± 5.43) years. The patient’s age, PSA, Gleason grade, PDL1 expression and other clinical data were collected for analysis. Patient inclusion criteria: (1) PCa diagnosed by pathological examination according to EAU-EANM-ESTRO-ESUR-SIOG Guidelines on Prostate Cancer (11); (2) There was no radiotherapy, chemotherapy, ADT and other treatments before operation; (3) There was no bone metastasis before surgery; (4) The patients and their families were informed of the study and signed the informed consent form. This study was approved by the Committee of the Army Military Medical University of China (approval no. 2019-YD-043-01) and was carried out in accordance with the Declaration of Helsinki. All methods were carried out as per relevant guidelines and regulations.

### Immunohistochemical staining and interpretation of results

A rabbit polyclonal antibody (pAb) specific for succinyllysine (PTM-401) was purchased from PTM Bio (Hangzhou, China); and immunohistochemical detection kits and DAB chromogenic reagent were purchased from Xinchao Company (Shanghai, China). The simple methods for IHC were as follows: the paraffin-embedded tissue paraffin block 3 μm thick serial sections were taken and routinely deparaffinized to water. The Multimer marker two-step assay was performed step-by-step by a fully automated multi-function pathology detection system (Benchmark GX, Roche). The basic conditions were: antigen retrieval for 30 min, washing with buffer, adding UV DAB inhibitor (Ultraview Universal DAB Detection Kit, Roche), 37°C for 4 min. Wash with buffer, add primary antibodies (PTM-401), and incubate at 37°C for 30 min. The buffer was washed, and UV HRP multimer (Ultraview Universal DAB Detection Kit, Roche) was added, and the temperature was 37°C for 8 min. The buffer was washed, and an equal volume of DAB and DAB H2O2 (Ultraview Universal DAB Detection Kit, Roche) was added, at 37°C for 8 min. Rinse with buffer, add Bluing Reagent, keep at 37°C for 4 min, rinse with buffer, and mount. Use PBS instead of the primary antibody as a negative control.

Tissue sections were repeatedly observed by 2 pathologists, and 5 high-power fields were randomly selected. According to the percentage of positive cells, the fields were scored as: 0 points for no positive cells, 1 point for the number of positive cells ≤ 10%, 2 points for 11% to 25%; 3 points for 26% to 50%; and 4 points for 51% to 100%. According to the intensity of positive staining, the fields were scored as: 0 points for no positive staining, 1 point for light yellow, 2 points for tan, and tan 3 points. The two scores are multiplied for the final score: high IHC scores ≥10, 10>medium IHC scores>6 and low IHC scores ≤6.

### Public data available

Transcriptome data and corresponding clinical data of 498 PCa patients were downloaded from the TCGA public database (TCGA-PRAD, https://www.cancer.gov/tcga). The samples with data annotation were removed in the original data process, and the average gene expression is lower than 1. According to the database information, the sequencing data were divided into the adjacent and PCa groups. After filtering and normalizing the data, a differential analysis was performed and RNA-seq data of 20 genes were associated with succinylation. Genes related to succinate metabolism include succinate dehydrogenase (SDHA, SDHB, SDHC and SDHD), succinyl-CoA ligase (SUCLA2, SUCLG1 and SUCLG2), protein post-translational modification related enzymes, erasure enzymes (SIRT1, SIRT2, SIRT3, SIRT4, SIRT5, SIRT6, SIRT7) and synthetases (CPT1A, CREBBP, GLYATL1, KAT5, MEAF6 and OXCT1).

### Statistical analysis

R software (https://www.r-project.org/) was used to analyze the data, and the correlation between the expression of anti-succinyllysine and the clinicopathological characteristics of the patients was analyzed by χ2 test or Fisher exact test. COX proportional hazards regression model was used to analyze the independent prognostic factors affecting pathological stage patients with PCa. P<0.05 denoted a statistically significant difference.

## Results

### Comparison of succinylation level between PCa and adjacent tissues

**Figure 1:**
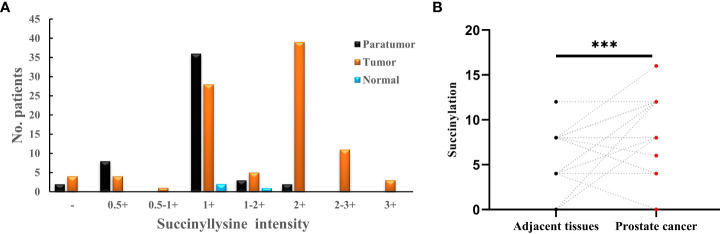
**(A)** Statistical analysis of anti-succinyllysine IHC staining distributed in prostate cancer, adjacent and normal prostate tissues; **(B)** Pair-wise analysis of anti-succinyllysine IHC scores between 52 pairs of prostate cancer and adjacent tissues. ***Statistically significant (p<0.001).

**Figure 2:**
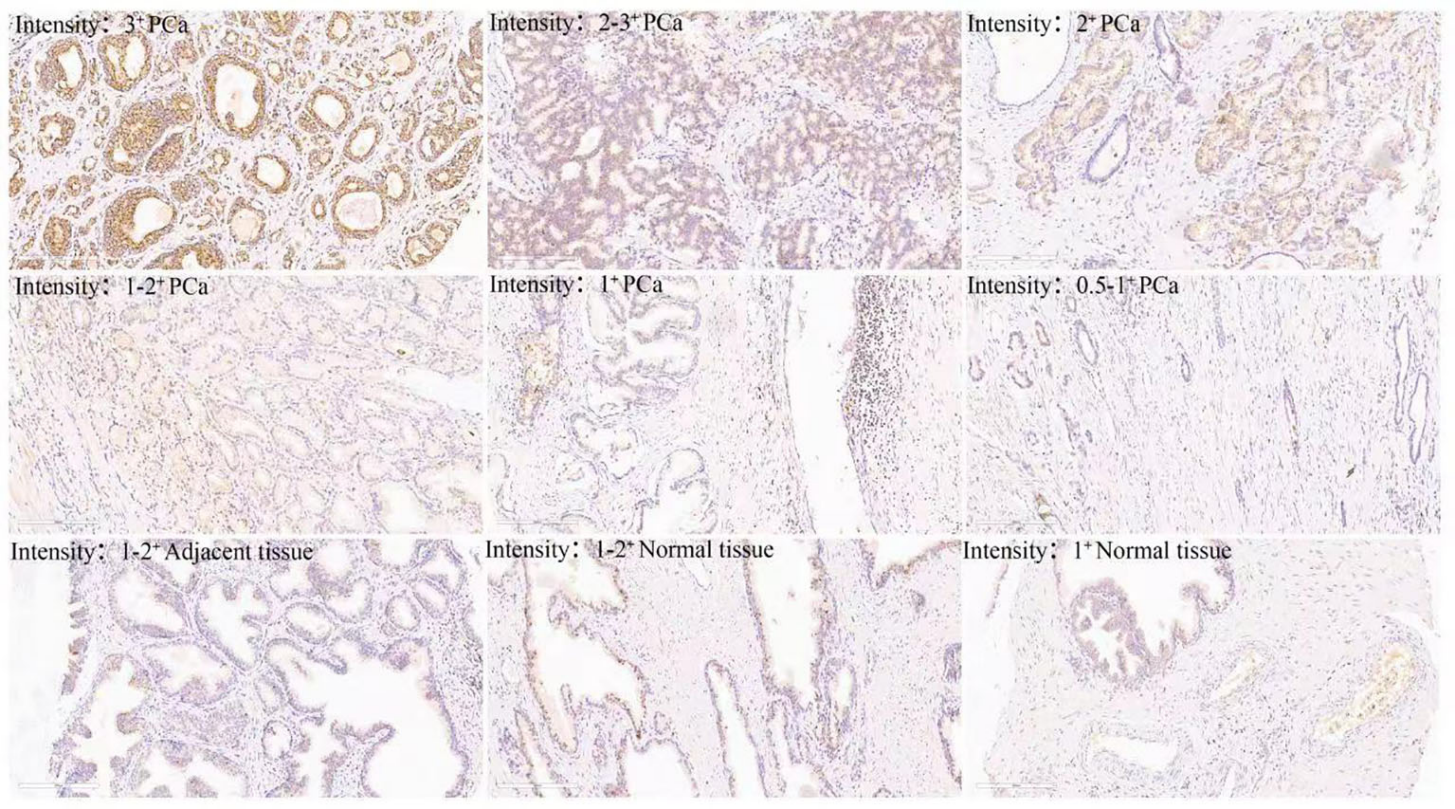
Different intensity of anti-succinyllysine IHC staining distributed in prostate cancer.

It has been reported that abnormal metabolism of succinate from the tumor environment has an anaplerotic effect that enhances the malignant potential of PCa cells (12). Succinate is the most important substrate for lysine succinylation modification. In this research, we first checked the expression of anti-succinyllysine among PCa, adjacent and normal prostate tissues. Compared with adjacent and normal prostate tissues, the level of anti-succinyllysine was higher in PCa (**Figure 1A** and **Table 1**, Fisher exact test, P<0.001). Regarding the location of succinylation, deep brown color is mainly distributed in the cytoplasm of basal and epithelial cells (**Figure 2**). In addition, a comparison of the level of succinylation in 52 pairwise PCa and adjacent tissues also demonstrated higher expression in PCa (**Figure 1B**). Therefore, high level of succinylation was identified in PCa prostate tissues, which may involve in the development of PCa.

**Table 1:** Differential expression of anti-succinyllysine in prostate cancer and adjacent tissues.

Variables	Anti-succinyllysine IHC scores			P value
	High	Medium	Low	
Prostate cancer (n=95)	53	33	9	<0.001
Adjacent tissues (n=52)	2	39	11	
Normal tissues (n=3)	0	3	0	

*Statistically significant (p<0.05).

Then, the whole anti-succinyllysine IHC scores were divided into high (IHC scores ≥10), medium (10>IHC scores>6) and low level (IHC scores ≤6) groups. Age distribution showed no significant difference among the three groups. However, the Gleason grades of III and IV are more enriched in the group of high IHC scores (**Table 2**, Fisher exact test, P<0.001). Several studies have confirmed that programmed cell death ligand 1 (PD-L1) targeting immune checkpoint inhibitors played an important role in the development of numerous tumors, and high PD-L1 expression was associated with poor clinical outcomes in patients with PCa (13). Therefore, we investigated the relationship between the level of succinylation and PDL1 expression. The results indicated the high level of PDL1 associated with high anti-succinyllysine IHC scores (**Table 2**, Fisher exact test, P<0.001).

**Table 2:** Correlation between succinyllysine expression and clinicopathological characteristics.

	Variables	Anti-succinyllysine IHC scores			P value
		High	Medium	Low	
	Age (year)	72.02 ± 6.22	69.15 ± 5.84	68.89 ± 6.29	0.23
Gleason	I	9	9	2	<0.001
	II	19	16	5	
	III	16	6	1	
	IV~	9	2	1	
PDL1	0	5	4	0	<0.001
	1	25	20	7	
	2~	21	5	1	

*Statistically significant (p<0.05).

### Risk model construction based on the expression of succinylation related genes

**Figure 3:**
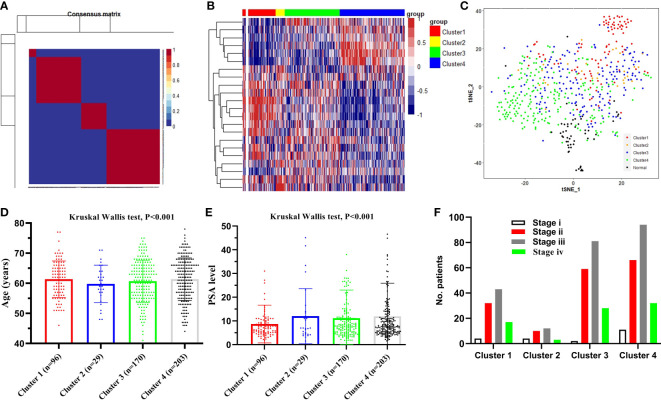
Identification of the sub-types of prostate cancer (PCa). **(A)** 498PCa samples were clustered into four types according to the consensus clustering matrix; **(B)** Heatmap plot shows 20 succinylation modification related genes were distributed in four clusters; **(C)** t-SNE analysis of 498 PCa and 52 adjacent samples based on expression levels of 20 succinylation modification related genes; **(D)** Comparison clinical characteristic of age among four clusters; **(E)** Comparison clinical characteristic of PSA level among four clusters; **(F)** Comparison clinical characteristic of pathologic stage among four clusters.

**Figure 4:**
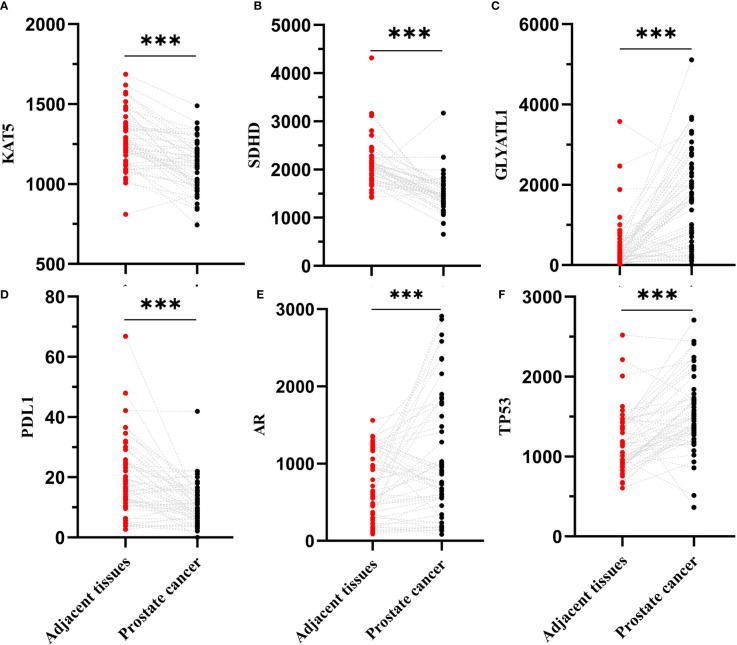
Comparison expression level of 6 succinylation modification related genes between 52 pairs of prostate cancer and adjacent tissues from TCGA database. **(A)** is KAT5; **(B)** is SDHD; **(C)** is GLYATL1; **(D)** is PDL1; **(E)** is AR; **(F)** is TP53. ***Statistically significant (p<0.001).

We hypothesized some gene expressions are associated with succinylation modification, and these genes dominate the progress of PCa. Therefore, RNA-seq data of 20 succinylation-related genes from the TCGA database were chosen for risk model construction. 498 PCa samples were clustered into four subtypes based on the RNA-seq data of 20 succinylation-related genes (**Figure 3A**) by using the NMF algorithm. The association matrix and expression differences among four subtypes were demonstrated in **Figures 3A, B**. In addition, four subtypes present differences distributed in the t-Distributed stochastic neighbor embedding (t-SNE) analysis of all patients, and the largest difference was demonstrated between clusters 1 and 3 (**Figure 3C**). More importantly, patients in cluster 4 are the highest in age (Kruskal Wallis test, P <0.001, **Figure 3D**) and PSA levels (Kruskal Wallis test, P <0.001, **Figure 3E**) compared with the other 3 clusters. But no pathological stage difference was found among the four clusters (**Figure 3F**). By comparing the expression level of 20 succinylation modification-related genes between 52 pairs of PCa and adjacent tissues from the TCGA database, we found three succinylation-related genes (KAT5, SDHD, and GLYATL1) that showed difference between the two groups (**Figure 4**). In addition, the expression of three prostate cancer-related genes differed in 52 pairs of tissues (PDL1, AR and TP53). In the six most different expression genes, GLYATL1 (P=0.005) and AR (P=0.04) expressions were associated with higher pathology stages by using multivariate linear regression analyses (**Table 3**).

**Table 3:** Multivariate liner regression analyses of the pathology stage of prostate cancer correlated with clinical characterizes and key genes.

	Estimate	Std.Error	t value	Pr(>|t|)
(Intercept)	2.2040	0.4109	5.36	0.0000
Age	0.0113	0.0049	2.30	0.02
PSA	0.0164	0.0028	5.903	0.00
KAT5	0.0010	0.0002	0.513	0.61
GLYATL1	0.0010	0.0000	2.83	0.005
TP53	0.0010	0.0001	1.45	0.15
AR	0.0010	0.0001	2.07	0.04
CD274	0.0040	0.0030	1.32	0.188
SDHD	0.0001	0.0001	0.57	0.57

*Statistically significant (p<0.05).

## Discussion

In summary, we found high levels of succinylation modification enriched in PCa patients compared with adjacent and normal prostate tissues. We provided evidence that succinylation modification-related genes contribute to the PCa pathological stage and may associate with the heterogeneity of PCa. We even found succinylation-related two genes (GLYATL1 and AR) that were also associated with higher pathology in PCa. However, the mechanism behind high levels of succinylation modification and the development of PCa remains to be explained.

Immunohistochemistry utilizes specific antibodies to detect and localize specific antigens in cells and tissues, which is an essential technique for tissue-based diagnostics and biomarker detection (14). It is usually detected and examined with a light microscope. Advances in technology have brought unprecedented sensitivity, specificity, and reproducibility to immunohistochemical analysis (15). In the human protein post-translational modification, succinylation modification is critical in many metabolic diseases and tumor progression (10, 16, 17). In the current study, we used pan-succinyllysine antibody to detect succinylation modification and the degree of antibody staining (18). Immunohistochemical experiments were performed using 95 cases of prostate cancer, 52 cases of adjacent tissue and 3 cases of normal control prostate paraffin-embedded tissue. The results confirmed that the succinylation was localized to the cytoplasm, with staining intensity ranging from pale yellow to tan. Succinylation modification is highly enriched in PCa tissues, and success modification is highly enriched in PCA tissues. More importantly, succinylation modification was significantly overexpressed in Gleason grades III and IV patients with prostate cancer. As we all know, Gleason grading system is a common method for pathological grading of PCa (19), and its score is closely related to the degree of malignancy, invasion, metastasis and prognosis of PCa (19). In this study, succinylation is highly expressed in patients with high Gleason grade, which suggests that the high expression of succinylation is closely related to the malignant degree of PCa, and succinylation is involved in the occurrence and development of PCa.

Programmed cell death receptor (PD-1) is an important immunosuppressive molecule, which can down-regulate the immune system response to avoid autoimmune diseases (20, 21). PD-1 receptor PD-L1 on the surface of tumor cells can bind to PD-1 on T cells, which promotes tumor cells’ immune escape. In addition, a recent study found that PD-L1 can bind DNA to control different pathways related to escaping immune surveillance or tumor microenvironment inflammation, thus participating in the occurrence and development of tumors (13, 22). As for PCa, studies have found that the high expression of PD-L1 is closely related to the adverse clinical outcomes of PCa patients. Blocking PD-1/PD-L1 signal transduction has been used in the clinical treatment of PCa (13, 22). In this study, we found that PDL1 was highly expressed in PCa, and there was a positive correlation between the high expression of PDL1 and high succinylation expression. Interestingly, the latest research shows that SIRT5 mediates desuccinylation modification and inhibits the metastasis of PCa (23). Therefore, combined with the literature and our findings, we speculate that succinylation-related signaling pathways could be involved in the immune escape, occurrence and development of PCa (13, 20–23).

Using public published big data, we found that the expression of succinylation-related gene Glycine-N-acyltransferase-like 1 (GLYATL1) and prostate cancer-related gene (AR) in 498 PCa tissues were significantly higher than that in non-cancer tissues. In addition, the expression levels of AR and GLYATL1 were correlated with the pathological stage. These findings suggest that AR and GLYATL1 were involved in the development of PCa. AR is the intersection of signal transduction systems in the growth, differentiation and malignant transformation of prostate epithelial cells, which is closely related to the occurrence and development of PCa (24–26). At present, AR is involved in the proliferation, differentiation, invasion, metastasis and immune escape of PCa (24–26).

There is currently no report about the important role played by GLYATL1 and succinylation. GLYAT in the detoxification of endogenous and exogenous acyl-CoA. GLYATL1 and GLYATL2 are members of the GLYAT family (27, 28), and GLYATL1 is not only involved in normal physiological metabolic activities in the human body, but also in tumorigenesis (27, 28). Studies suggest that the high expression of GLYATL1 is related to the improvement of overall survival of renal clear cell carcinoma, liver cancer, bladder cancer, cervical cancer, lung cancer, ovarian cancer and other tumors (27, 28). In PCa, GLYATL1 is highly expressed, and low-grade PCa has higher glyatl1 expression than high-grade PCa (29, 30). At the same time, the study found that the knockout of GLYATL1 gene in PCa significantly inhibited the colony formation ability of LNCaP cells (29, 30). In this study, we found that the expression level of succinylation related gene GLYATL1 was closely related to the TNM stage of PCa (P < 0.05). To sum up, we believe that the expression level of GLYATL1 is closely related to the occurrence and development of PCa and GLYATL1 mRNA may become one of the molecular markers for evaluating PCa succinylation modification, but this conclusion still needs to be further confirmed.

Despite the numerous findings of this study, it has limitations. First of all, the number of samples involved in this study is small. In the future, a greater amount of sample is needed to further confirm the research results. Second, this study did not elaborate the molecular mechanism in depth, and the role of success in prostate cancer needs to be further elaborated in the future. Third, part of the data in this study comes from biological information analysis, and the analysis itself has bias limitations. Fourth, because of differences in race, age and disease progression, the research results are not representative enough.

## Conclusion

This study found that the level of succinylation in PCa tissue was significantly increased, and high level of succinylation was associated with high Gleason grade and high PDL1 expression in PCa. The model construction of 20 succinylation related genes showed that the succinylation related gene GLYATL1 might be closely related to the late pathological stage of PCa.

## Data availability statement

The data analyzed in this study is subject to the following licenses/restrictions: The datasets generated and analyzed during the current study are not publicly available due to health privacy concerns, but are available from the corresponding author on reasonable request. Requests to access these datasets should be directed to urologyzhaoj@sohu.com.

## Ethics statement

This study was approved by the Committee of the Army Military Medical University of China (approval no. 2019-YD-043-01). The patients/participants provided their written informed consent to participate in this study.

## Author contributions

ZZ, JZ, SD: Conceptualization, methodology, investigation, writing - original draft. YC, LF: Investigation, software. ZZ, JZ, SD: Investigation, validation, resources. ZZ, JZ, SD: Formal analysis, data curation, visualization, writing - review and editing, funding acquisition, supervision. All authors contributed to the article and approved the submitted version.

## Funding

This study was supported by the National Natural Science Foundation of China (NSFC81974101).

## Conflict of interest

The authors declare that the research was conducted in the absence of any commercial or financial relationships that could be construed as a potential conflict of interest.

## Publisher’s note

All claims expressed in this article are solely those of the authors and do not necessarily represent those of their affiliated organizations, or those of the publisher, the editors and the reviewers. Any product that may be evaluated in this article, or claim that may be made by its manufacturer, is not guaranteed or endorsed by the publisher.
